# A pragmatic multi-setting healthy lifestyle intervention to improve BMI status in a middle-income population: A potential strategy for individuals at risk

**DOI:** 10.34172/hpp.43196

**Published:** 2024-12-30

**Authors:** Reza Yari-Boroujeni, Leila Cheraghi, Parnian Parvin, Fatemeh Shiravi, Hasti Masihay-Akbar, Amirabbas Momenan, Arash Ghanbarian, Parvin Mirmiran, Davood Khalili, Fereidoun Azizi, Parisa Amiri

**Affiliations:** ^1^Research Center for Social Determinants of Health, Research Institute for Metabolic and Obesity Disorders, Research Institute for Endocrine Sciences, Shahid Beheshti University of Medical Sciences, Tehran, Iran; ^2^Department of Epidemiology and Biostatistics, Research Institute for Endocrine Sciences, Shahid Beheshti University of Medical Sciences, Tehran, Iran; ^3^Prevention of Metabolic Disorders Research Center, Research Institute for Endocrine Sciences, Shahid Beheshti University of Medical Sciences, Tehran, Iran; ^4^Nutrition and Endocrine Research Center, Research Institute for Endocrine Sciences, Shahid Beheshti University of Medical Sciences, Tehran, Iran; ^5^Endocrine Research Center, Research Institute for Endocrine Sciences, Shahid Beheshti University of Medical Sciences, Tehran, Iran

**Keywords:** Body mass index, Developing countries, Health promotion, Obesity, Quantile regression analysis

## Abstract

**Background:**

To evaluate a multi-setting lifestyle intervention’s effect on body mass index (BMI) across the entire spectrum in a middle-income adult population over 15 years.

**Methods:**

This pragmatic interventional study included 5153 adults (≥20 years) from a middle-income community, followed for over 15 years with five follow-ups. A multi-setting intervention (school, family, community) aimed to promote healthy lifestyles. The lambda-mu sigma (LMS) method and quantile regression model were used to analyze changes in BMI percentiles (10^th^-90^th^) by sex and intervention group.

**Results:**

The intervention showed modest effects on BMI percentiles. In men, it lowered BMI at the 40^th^ and 70^th^ percentiles (overweight/obesity onset) at the first follow-up (β=-0.16, 95% CI: -0.33, -0.001 and β=-0.21, 95% CI: -0.38, -0.04 respectively). In women, the effect emerged later (second follow-up) at the 20^th^ (β=-0.39, 95% CI: -0.60, -0.18), 30^th^ (β=-0.27, 95% CI: -0.49, -0.04), and 60^th^ (β=-0.20, 95% CI: -0.39, -0.02) percentiles (overweight risk), extending to more overweight percentiles (20^th^-50^th^) in the third follow-up (βs ranged from -0.28 until -0.26).

**Conclusion:**

Our results indicated the effectiveness of a practical lifestyle intervention to control rising trend of BMI at the onset of overweight and obesity in a middle-income population. These findings can be useful for planning obesity prevention programs in communities with similar socioeconomic statuses.

## Introduction

 The global deaths and disability-adjusted life years (DALYs) related to high body mass index (BMI) doubled over the past three decades.^1^ Currently, 39% of the world’s adult population is overweight, and 13% is obese.^2^ This trend is particularly concerning in low and middle-income countries, where 48.2% of the population has a BMI higher than expected.^3,4^ Although the Middle East has not seen a significant rise in overweight and obesity rates over the last twenty years, the region still reports higher than other low/middle-income countries, with 33.14% of the population being overweight and 21.17% being obese.^5^ Iran, as a low/middle-income country, mirrors these regional trends, with similar percentages of obese and overweight adult population.^5,6^

 The widespread global prevalence of obesity/overweight has drawn much attention to feasible and cost-effective interventions to mitigate its physical and psychosocial consequences at various individual and community levels.^7-10^ The goal of these interventions is to ensure long-term healthy weight maintenance. Although individual-based interventions such as healthy diets, increased physical activity, and anti-obesity medications can effectively balance daily energy intake and expenditure,^11^ they often focus more on weight loss than on the primary prevention of obesity and are typically accessible only to individuals with higher socio-economic status.^12^ Additionally, these interventions frequently fail to support sustained weight maintenance and tend to be costlier per kilogram of weight reduction compared to community-based approaches.^11,12^ Hence, increasing social support and public awareness through community-based interventions is recognized as an efficient and cost-effective strategy for obesity prevention, especially in developing countries with limited health resources.^13^

 Limited research exists on the long-term effects of community-based weight management interventions in low/middle-income countries. Recently, a systematic review has shown that these interventions can have positive results in these countries similar to those observed in some developed countries.^14^ However, fundamental differences in healthcare systems and cultural contexts between societies with varying socio-economic status raises serious doubts about the feasibility of implementing similar interventions in the aforementioned countries.^15^ Moreover, previous studies often overlooked initial weight differences among individuals, leading to varied perceptions of obesity threats and lifestyle modification benefits, which can play a decisive role in achieving the expected goals.^16^ To the best of our knowledge, only one longitudinal study conducted in the United States from 1970 to 2006, has examined the effect of a community-based intervention on different weight percentiles, showing effectiveness at higher BMI percentiles.^17^

 Iran, a low/middle-income country undergoing urbanization and nutritional transition,^18^ requires tailored and feasible community-based interventions to promote healthy lifestyles and weight management. No specific study has been found on the distribution of BMI percentiles in Iran. The Tehran Lipid and Glucose Study (TLGS) is a community-based lifestyle intervention program aimed at preventing non-communicable diseases (NCDs) and their risk factors. Based on a previous study, lifestyle interventions in the TLGS adult population can reduce cardiovascular disease risk factors in the short-term.^19^ The current study is the first report of the TLGS that indicates the effectiveness of a pragmatic multi-setting healthy lifestyle intervention on full-spectrum of BMI in men and women over more than fifteen-year follow-up period.

## Material and Methods

###  Study design 

 The TLGS is an ongoing cohort study initiated in 1999, focusing on tracking NCDs and their risk factors in an urban population. After collecting baseline data in 2002, the study incorporated an interventional phase conducted within TLGS participants. This phase, implemented through multi-settings (school, family, community), focused on evaluating the effectiveness and feasibility of lifestyle modifications in preventing or delaying the onset of NCD risk factors in the intervention group compared with the control group.

###  Inclusion/exclusion criteria and sample size

 TLGS included participants aged three years and older, regardless of sex, with consent from all family members, including those without any risk factors. Participants had to be residents of District 13 in Tehran, Iran. District 13 was selected due to its stable population, generalizability in terms of age and socioeconomic status, and the availability of extensive family data. The study excluded individuals with mental disabilities. In 1999, the baseline sample size for the TLGS study was set at 14 280, accounting for predicted dyslipidemia prevalence, a 95% confidence interval, 80% study power, 20% attrition rate, and a 2% design effect across seven age groups and both sexes. Over 15 000 individuals (aged 3-80) participated in the study, which used a multistage cluster random sampling approach. This involved selecting three out of twenty healthcare centers in the district, followed by the random selection of the populations served by these centers for participation. After collecting baseline data in 2002, to evaluate the effectiveness and feasibility of lifestyle modifications, a sample size of 4750 was calculated for the intervention group, based on factors such as a 5% reduction in serum cholesterol, two genders, three age groups, a design effect equal to 1.05, and a 30% attrition rate. One of the three centers, located further away and with a sufficient number of participants, was designated as the intervention site, where a multi-setting healthy lifestyle program was implemented. The other two centers served as control groups, receiving standard national healthcare. Both groups were assessed every three years.^20^

**Figure 1:**
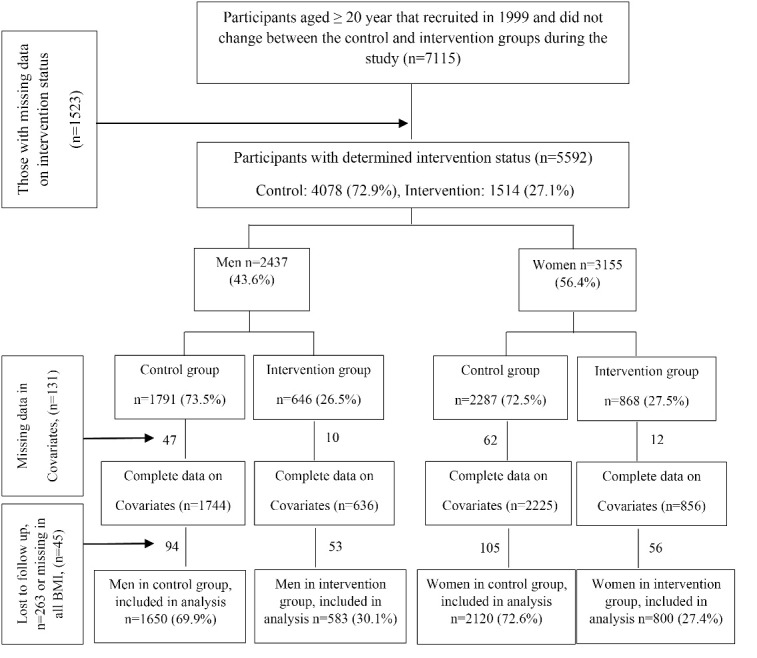
The flow diagram of the study

 For the current analysis, we focused on adult participants (aged ≥ 20 years) who were followed for five subsequent follow-ups until 2017. Of the 5592 participants at baseline, 131 were excluded due to missing data on covariates. Further, 263 participants did not complete the follow-up, and 45 had no BMI data in any follow-ups, resulting in a final analytic sample of 5153 participants (1383 intervention and 3770 control). Response rate was 91.3% and 92.4% for intervention and control groups respectively. Further details of the current sampling design are illustrated in Figure 1.

###  Intervention

 The TLGS healthy lifestyle intervention was conducted in three settings (community, family, and schools) in the intervention area. It was aimed to target three main aspects of lifestyle: nutrition, physical activity, and smoking. The intervention components for each setting were designed by the TLGS scientific committee and based on American Heart Association guidelines, the North Karelia project, and the baseline Knowledge, Attitude, and Practice (KAP) study.^21-23^ The trained health volunteers (health liaisons) were the main pillar for intervention implementation and responsible for inviting families, recruiting participants, organizing community events, coordinating with school liaisons, and distributing educational materials. The intervention health care center supervised them. The details of the context of the intervention in each setting are briefly described below^24^:

####  Community 

 The TLGS intervention engaged various public sectors, including healthcare centers, mosques, local amphitheaters, and conference halls, to deliver community components. Two to four conferences were held yearly during religious ceremonies, the holy month of Ramadan, and global occasions like World No Tobacco Day and World Diabetes Day. Nutrition education was delivered through face-to-face consultations, films, and slides, accompanied by distributed pamphlets, covering food groups, types of fats, recommended portions by age, and guidelines for reducing fat in foods and preparing healthy recipes. Key nutrition concepts included preparing low-fat foods, high-fiber diets, reducing frying, increasing fruit and vegetable intake, and also avoiding fast foods and unhealthy snacks. A quit smoking clinic has been set up to assist individuals in overcoming smoking addiction. General practitioners at the clinic provide support through face-to-face consultations and distribute pamphlets and brochures to help patients quit smoking. More than 80% of participants attended at least one of these events between every two follow-ups.

####  Families

 Health-related behaviors were targeted in the family context to boost the intervention’s effectiveness. Quarterly journal “courier of health”, booklets or pamphlets were published two to four times a year and distributed to families in the intervention area, covering topics like food pyramid, balanced diet, active living, health hazards of smoking, and smoking cessation techniques. Telephone surveys showed that all households received educational materials, and at least half of them read and paid attention to the content.

 Moreover, the health liaisons invited families to participate in group sessions. The sessions were two hours of face-to-face talk and a video/slide show with approximately 20 attendees. The central theme of the meetings was how to lead a healthy lifestyle by cooking and eating healthy food, being physically active, and living tobacco-free. Smokers were identified, encouraged to quit, and referred to cessation clinics. In addition, Stress control educational sessions were conducted, advisory stress clinics were held, and written brochures and booklets related to coping with stress were distributed.

####  Schools

 In the intervention area, 12 schools were designated as “Health Promoting Schools” to minimize contamination from the control area. These schools featured an enhanced health curriculum, which included the Living Tobacco-Free program with four main components: classroom curriculum and practice, student activities in the school anti-tobacco society, anti-tobacco policies in school, and family (parents) involvement. All principals and volunteer teachers were trained by physicians to enable the team to educate schoolchildren, covering smoking prevalence and its short-term and long-term consequences, the psychology of coping with stress, and strategies for quitting smoking. A nutrition educational program was introduced, which included lessons that paralleled the information offered in the children’s curriculum. The objective was to introduce food groups based on the food guide pyramid and nutrition concepts such as trying low-fat foods and dairies, high fiber diets, and healthy snacks, with red and green labels distinguishing unhealthy and healthy foods. In addition, the importance of physical activity was proclaimed, and morning exercises, theoretical and practical training for schoolchildren were provided. Children were encouraged to become members of a school sports team, and sport competitions were arranged as part of the intervention components in the schools.

 Every year, health classes were held for new-coming 7th graders and those who failed the previous class. Peer teaching was a prominent component of school-based education. Volunteer students formed a “school health society” and were responsible for teaching healthy living and acting as a role model for their classmates. Parents were also targeted in the school setting. They were involved through regular teacher/parent meetings and annual two-day seminars followed by 45-minute Q&A sessions, and educational materials. Evaluation surveys indicated that although some families refused to participate in related programs, almost 70% of planned school-based interventions were successfully implemented.

###  Measurements

 In each follow-up examination, trained interviewers obtained participants’ socio-demographics (age, sex, education, marital experience, employment) and behavioral information (smoking and physical activity) via validated questionnaires. Education was categorized as less than a high school diploma ( < 12 years of education), and high school diploma or higher ( ≥ 12 years of education). Employment was categorized based on employed or unemployed. Regarding marital status, the participants were divided into two groups with a history of marriage and no marriage experience in the past. The WHO adult smoking questionnaire (GAT) assessed smoking, and those who said yes or occasionally to the question “do you currently smoke?” was classified as a current smoker.^25^ This study assessed physical activity at baseline using the Lipid Research Clinics (LRC) questionnaire. This questionnaire examines physical activity in three sub-scales: regular, strenuous, and self-rated physical activity. In this study, the results were classified into two categories: high (at least three times a week) and low (less than three times a week).^26^

###  Primary outcome

 The outcome of the current study is BMI which is considered as its whole spectrum using different percentiles. BMI was calculated as weight divided by squared height (kg/m^2^). The trained general physician conducted physical examinations. As a part of the physical examination, participants’ weight was measured using a digital scale (Seca 707: range 0–150 kg) after removing shoes and heavy clothing. The weight of the participants was measured with an accuracy of 100 g. Height was measured by a tape meter stadiometer and without shoes while their head, shoulders, buttocks, and heels were touched the wall. Based on WHO cut-off points in adults, BMI between 25 and 30 kg/m^2^ and BMI ≥ 30 kg/m^2^ were defined as overweight and obesity, respectively.^27^

###  Statistical analysis

 Baseline characteristics of participants were described as mean × SD for continuous and frequencies (%) for categorical variables and were compared between the intervention and control groups using independent samples *t* test and the chi-square test, respectively. For men and women and for each study group, the age-specific curves of different percentiles (10%, 20%, 30%, 40%, 50%, 60%, 70%, 80%, and 90%) of individual BMI distribution at baseline and five follow-up examinations were presented using the lambda-mu-sigma (LMS) method in the Vector Generalized Linear and Additive Models (VGAM) package R version 2.15.1 (R Development core team, Vienna Austria).^28^ The quantile regression model as a robust statistical method was used to evaluate intervention effect on different BMI percentiles at baseline and during five follow-up examinations.^29^ Using this approach, it is possible to generalize the effectiveness of the lifestyle intervention on the whole distribution of BMI, especially the mentioned percentiles. The results of this model for each percentile (reported as βs) represent the difference in the value of BMI at that percentile for intervention compared to the control group. The models were adjusted for baseline assessment of BMI (except for comparing the percentiles at baseline), education, occupation, marital status, and also age for each follow-up. The statistical analysis was performed using the R software version 2.15.1 (R Development core team, Vienna Austria) and the significance level was considered as alpha = 0.05.

## Results

###  Comparing baseline characteristics between intervention and control groups 

 Data on 5153 adults (43.3% men) who participated in the TLGS from 1999-2001 were recruited in the current analysis and followed for a median of 15.8 years (Q1 = 14.7 years and Q3 = 16.7 years). Table 1 shows participants’ baseline characteristics in intervention and control groups. For both men and women, there was a significant difference in the level of education (*P* < 0.001 and *P* = 0.002, respectively) and smoking (*P* = 0.002 and *P* = 0.007, respectively). However, physical activity and marital experience in women (*P* = 0.001 and *P* = 0.003, respectively) and age only in men (*P* = 0.003) were significantly different among the control and intervention groups.

###  Changes in percentile BMI curves in men and women over study assessments 

**Figure 2:**
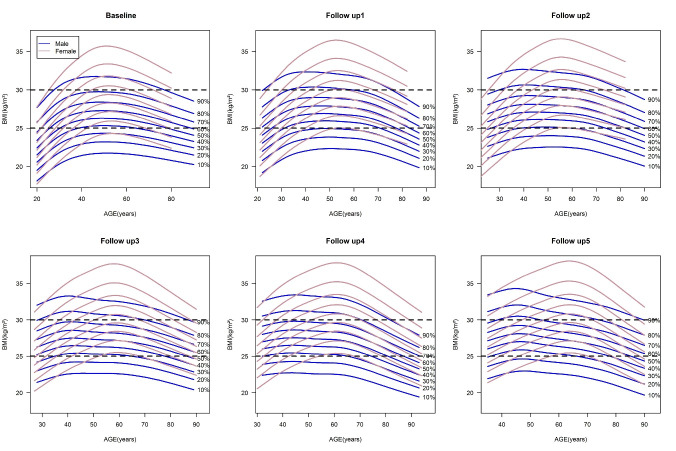
BMI percentile curves in baseline and during five follow-up examinations for both sexes. The curves from bottom to top represent the 10^th^ to 90^th^ percentiles for both sexes, respectively. Horizontal dash lines represent overweight and obesity cut-offs

Figure 2 shows the 10^th^ to 90^th^ percentile BMI curves by age and sex at baseline and five follow-up examinations. Horizontal dash lines represent overweight and obesity cut-offs. Although the BMI percentile curves showed increasing trends from baseline to the last follow-up in both sexes, women were placed upper than men in all assessment times. This median difference became more significant over time. All BMI percentiles in men started to decrease at the beginning of the fifth decade of life, but in women, this decreasing trend was observed more steeply in the sixth decade of life. In addition, for both sexes, the main characteristic was that each percentile moved away from the lower part of the box (lower levels of BMI) and approached the box ceiling (higher levels of BMI) over time. Therefore, a significant part of the individuals has gone out of the normal range of BMI during the study assessments.

###  Age-specific changes in percentile BMI curves among intervention and control groups over study assessments for both sexes

**Figure 3:**
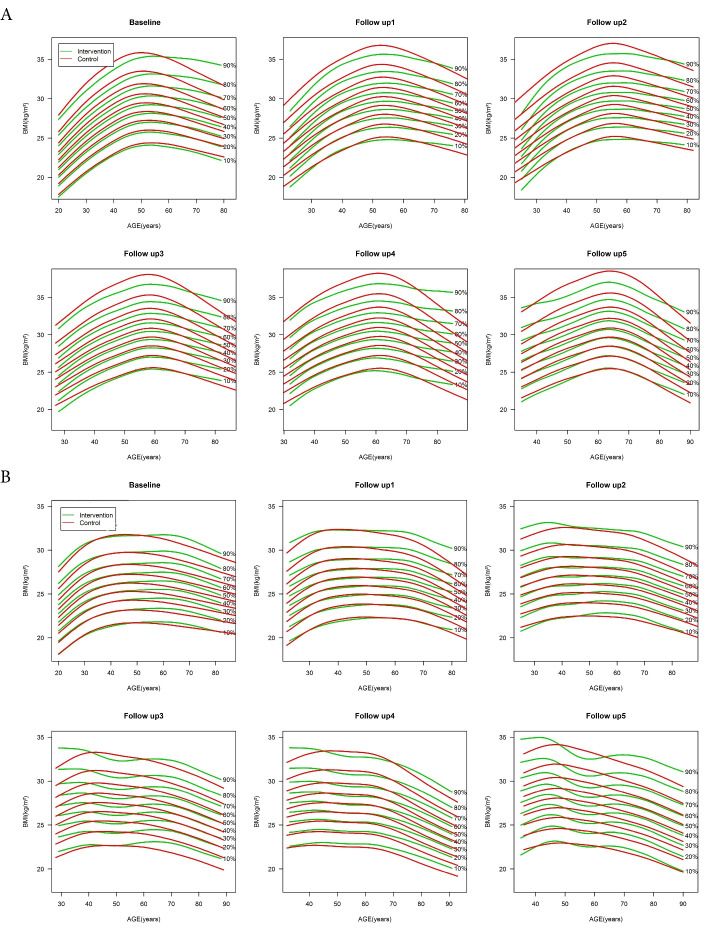
Age-specific BMI percentile curves in intervention and control groups in baseline and during five follow-up examinations for women (A) and men (B). The curves from bottom to top represent the 10^th^ to 90^th^ percentiles for both sexes, respectively. Horizontal dash lines represent overweight and obesity cut-offs

Figure 3 shows the age-specific BMI percentile curves in intervention and control groups at baseline and five follow-up examinations for both sexes. In women, upper BMI percentiles were different between the intervention and control groups at baseline and all follow-up assessments regardless of their age. Furthermore, in women participants approximately aged < 65 years, the upper percentiles of BMI were higher in the control group, which grew throughout the follow-up assessments. Among older women, the upper percentiles of BMI were higher in the intervention group at baseline, which gradually decreased during the subsequent two follow-up periods. This result was not observed in the later follow-ups. The current results showed no difference in BMI percentiles between study groups at baseline for middle-aged men. However, compared to the intervention group, BMI was higher in the control group during the latest follow-ups, especially in its upper percentiles. Further illustrations of Figure 2 revealed that the BMI percentile curves were placed higher for intervention than the control group in men of other ages.

## The effect of lifestyle intervention on different BMI percentiles over study assessments

Table 2 shows the estimated coefficients for the quantile regression model for men and women at baseline and each follow-up examination. Except for the 70^th^ BMI percentile in women (β = -0.481, *P* = 0.03), and the 90^th^ BMI percentile in men (β = 0.674, *P* = 0.02), none of the others differed between the intervention and control groups significantly in both sexes at baseline. Compared to men in the control group, those who participated in the intervention, the 40^th^ and 70^th^ BMI percentiles were significantly lower at the first follow-up examination (β = -0.164, *P* = 0.04 and β = -0.207, *P* = 0.01 respectively). In women, the effect of lifestyle intervention was significant in some of the second (the 20^th^, 30^th^, and 60^th^), third (the 20^th^, 30^th^, 40^th^, and 50^th^), and fifth (the 20^th^, 80^th^) follow-up examination percentiles.

 The results of the non-respondent analysis are presented in Table 3. Although BMI, physical activity, education, and marital experience were not different (*P* = 0.84, *P* = 0.08, *P* = 1.00, and *P* = 0.74, respectively), other baseline characteristics differed significantly.

**Table 1:** Descriptive statistics of participants in intervention and control groups at baseline

	**Men (n=2233)**			**Women (n=2920)**		
	**Control** **(n=1650)**	**Intervention** **(n=583)**	***P*** ** value**	**Control** **(n=2120)**	**Intervention** **(n=800)**	***P*** ** value**
Age(year)	43.59±15.12	46.92±16.08	0.003	41.71±13.78	42.78±13.95	0.065
Education			< 0.001			0.002
Less than higher school diploma	683(41.4)	303(52.0)		1193(56.3)	502(62.8)	
High school diploma and higher	967(58.6)	280(48.0)		927(43.7)	298(37.3)	
Employment			0.023			0.587
Employed	1197(72.5)	394(67.6)		213(10.0)	75(9.4)	
Unemployed	453(27.5)	189(32.4)		1907(90.0)	725(90.6)	
Marriage experience			0.391			0.003
Yes	1327(80.4)	479(82.2)		1879(88.6)	676(84.5)	
No	323(19.6)	104(17.8)		241(11.4)	124(15.5)	
Physical activity			0.191			0.001
Low	1283(78.2)	440(75.6)		1503(71.1)	615(77.2)	
High	357(21.8)	142(24.4)		610(28.9)	182(22.8)	
Smoking			0.002			0.007
Yes	439(26.8)	118(20.3)		64(3.0)	10(1.3)	
No	1202(73.2)	464(79.7)		2050(97.0)	788(98.7)	
BMI (kg/m^2^)	25.65±4.07	25.93±4.07	0.488	27.65±4.95	27.43±4.89	0.453

Values are Mean (SD) for continuous variables and n (%) for categorical variables. The *P* value is for comparison between intervention and control groups in each gender population.

**Table 2:** The effects of lifestyle education on different BMI percentiles over five follow-up examinations: the quantile regression results

**Coefficient** ^a^ ** (SE) **									
	**10** ^th^	**20** ^th^	**30** ^th^	**40** ^th^	**50** ^th^	**60** ^th^	**70** ^th^	**80** ^th^	**90** ^th^
**Men**									
Baseline	0.264 (0.291)	0.237 (0.244)	0.123 (0.221)	0.125 (0.233)	0.283 (0.244)	0.376 (0.207)	0.206 (0.281)	0.518 (0.318)	0.674 (0.298)*
Follow up 1	-0.209 (0.117)	-0.097 (0.115)	-0.141 (0.084)	-0.164 (0.083)*	-0.111 (0.084)	-0.132 (0.085)	-0.207 (0.087)*	-0.056 (0.107)	-0.221 (0.132)
Follow up 2	-0.068 (0.159)	-0.018 (0.127)	0.038 (0.102)	0.020 (0.111)	-0.004 (0.090)	-0.054 (0.100)	-0.035 (0.107)	-0.180 (0.144)	-0.055 (0.150)
Follow up 3	0.056 (0.209)	0.019 (0.143)	-0.097 (0.126)	-0.078 (0.103)	-0.076 (0.122)	-0.029 (0.117)	-0.120 (0.129)	-0.073 (0.135)	-0.384 (0.203)
Follow up 4	-0.013 (0.200)	-0.062 (0.181)	-0.135 (0.159)	-0.091 (0.135)	-0.110 (0.139)	-0.157 (0.135)	-0.125 (0.175)	-0.116 (0.175)	-0.124 (0.270)
Follow up 5	-0.094 (0.236)	-0.167 (0.173)	-0.048 (0.175)	0.016 (0.192)	0.122 (0.196)	0.213 (0.167)	-0.007 (0.176)	-0.133 (0.219)	-0.143 (0.298)
**Women**									
Baseline	-0.128 (0.226)	-0.274 (0.226)	-0.083 (0.223)	-0.176 (0.229)	-0.022 (0.217)	-0.243 (0.225)	-0.481 (0.222)*	-0.579 (0.309)	-0.452 (0.372)
Follow up 1	-0.076 (0.177)	-0.073 (0.101)	-0.003 (0.095)	0.045 (0.087)	0.040 (0.075)	0.048 (0.093)	0.060 (0.106)	0.149 (0.121)	0.207 (0.163)
Follow up 2	-0.240 (0.167)	-0.392 (0.107)*	-0.269 (0.115)*	-0.101 (0.113)	-0.104 (0.095)	-0.204 (0.094)*	-0.147 (0.115)	-0.202 (0.127)	-0.330 (0.198)
Follow up 3	-0.213 (0.163)	-0.280 (0.127)*	-0.257 (0.132)*	-0.258 (0.125)*	-0.281 (0.119)*	-0.228 (0.126)	-0.199 (0.162)	-0.097 (0.142)	-0.087 (0.276)
Follow up 4	-0.161 (0.208)	-0.030 (0.192)	0.003 (0.126)	-0.122 (0.129)	-0.152 (0.149)	-0.035 (0.153)	-0.172 (0.131)	-0.205 (0.165)	-0.299 (0.312)
Follow up 5	-0.353 (0.246)	-0.409 (0.184)*	-0.130 (0.172)	-0.161 (0.169)	0.162 (0.165)	-0.129 (0.155)	-0.122 (0.180)	-0.450 (0.202)*	-0.275 (0.289)

Baseline assessment was conducted from 1999 to 2001. The subsequent follow-ups were conducted 3, 6, 9, 12, and 15 years after the baseline assessment. ^a^ The coeficient represents the differene in the value of BMI at the n^th^percentile for intervention compare to control group over study assesment. SEs represents standard errors. The models were adjusted for BMI, education, employment, marital experience at baseline, and age for each follow-up. * *P* value < 0.05.

**Table 3:** Comparison of baseline characteristics among respondent and non-respondent participants

	**Non-responders** ^a^ **(n=1962)**	**Responders** **(n=5153)**	***P*** ** value**
Age (year)	43.95 ± 16.76	43.06 ± 14.60	0.028
Education			1.000
Less than higher school diploma	1014 (52.0)	2681 (52.0)	
High school diploma or higher	936 (48.0)	2472 (48.0)	
Employment			< 0.001
Employed	622 (31.8)	1879 (36.5)	
Unemployed	1335 (68.2)	3274 (63.5)	
Marital experience			0.740
Yes	1667 (85.0)	4361 (84.6)	
No	295 (15.0)	792 (15.4)	
Physical activity			0.089
Low	1413 (76.8)	3841 (74.8)	
high	426 (23.2)	1291 (25.2)	
Smoking			0.002
Yes	421 (22.9)	998 (19.4)	
No	1421 (77.1)	4136 (80.6)	
**BMI (kg/m** ^ 2 ^ **)**	26.80 ± 4.97	26.78 ± 4.67	0.841

^a^ Participants who did not come back at follow-ups.

## Discussion

 This study aimed to evaluate the sex-specific effectiveness of a pragmatic multi-setting lifestyle intervention on the full spectrum of BMI in a middle-income community through a fifteen-year follow-up. Our results showed that in a sex-specific pattern, the current intervention in the short term improves BMI status in participants at risk of gaining excess weight and obesity. In this regard, the effect of the current intervention on the BMI in men who were transitioning to overweight (40^th^ percentile) or obesity (70^th^ percentile) was observed in short term (the first follow-up examination). Also, in women with similar BMI transition (20^th^-30^th^ percentiles and 60^th^ percentile for overweight and obesity, respectively), the effect of the intervention was observed at a later time (the second follow-up examination), which was extended to all overweight women (20^th^ to 50^th^ percentiles) in the subsequent follow-up. However, the mentioned effectiveness of the intervention did not sustain in the long term.

 Investigating the effect of healthy lifestyle intervention on different BMI percentiles in a middle-income community is a unique feature of this study. Based on findings, the effect of such interventions were mainly observed in men and women on the onset of being overweight or obese, particularly around BMI levels of 25 and 30. Although no significant difference was observed in the physical activity level and energy intake in previous TLGS studies between intervention and control groups, current smoking prevalence differed at all follow-ups.^30,31^ It is reasonable to hypothesize that individuals who change their BMI classification are more likely to make healthy decisions, so investigating each of these factors related to BMI percentiles in future studies is recommended. The effectiveness of the lifestyle intervention in this study has disappeared in the long term. Although similar studies have conducted interventions and follow-ups in a shorter period, it has been declared that a return to the pre-intervention BMI can be expected in the absence of intervention continuation.^32^

 The current lifestyle intervention study aimed to be practical and scalable within the existing societal structures of a middle-income country with limited resources. Based on the obtained results, while the significant effects of the intervention on weight control of both sexes were evident in the first follow-up evaluation, the stability of this effect was longer in women than in men. Research indicates that men often have inaccurate perceptions and dissatisfaction with their weight across the BMI spectrum, leading to less effort in weight loss and program participation.^33-35^ On the other hand, women, who typically manage family health and actively engage with and disseminate health information, are expected to be more positively impacted by such interventions.^36,37^

 Promoting healthy lifestyles through community awareness has always been one of the most practical and cost-effective strategies for obesity prevention, especially in low/middle-income countries. Therefore, healthy lifestyle education has a major contribution in planned community-based interventions. Since lifestyle education is mainly based on the transmission of health information, an individual’s willingness to acquire this information plays an important role in the effectiveness of such interventions. According to Figure 3, the BMI improvement in the intervention group, compared to the control group, is mainly observed in the middle-aged and elderly participants, especially in the upper percentiles. The results of previous studies showed that these age groups exhibit more protective health behaviors and seek health information more than younger individuals.^38-40^ On the other hand, in line with other studies,^41-43^ movement of BMI percentiles towards higher values mainly occurs in younger age groups. Factors such as entering the job market, reduced leisure time, less physical activity, dietary responsibility, preference for processed foods, and lower interest in health information could led to inappropriate lifestyle changes.^44^ This suggests that although obesity is more prevalent in middle age, it often stems from poor lifestyle choices made in early adulthood.

 Our study, consistent with national data,^6^ shows a general increase in BMI over time, largely due to urbanization-related lifestyle changes such as altered diets and reduced physical activity during the past decades.^45-48^ In the current study, while both sexes experience an increasing of BMI trend, this increase is more remarkable in women. This sex-specific pattern confirms previous findings in Iran,^6,49^ and other middle-income countries,^13,50-52^ suggesting behavioral,^53,54^ cultural,^55-57^ social,^58,59^ and biological^60-64^ reasons. In addition, women experience a longer period of increasing BMI, extending into their sixth decade of life compared to the fifth decade in men; although in both sexes, it has ended with a downward trend in the elderly individuals. This gender and age-specific pattern is consistent with findings from other studies in Iran,^6^ and other countries as well.^65-67^

 This study has both strengths and limitations. As a large population-based study conducted in a middle-income community in Middle East, the current study provided a unique opportunity to determine the effect of a multi-setting practical lifestyle intervention on the full spectrum of BMI in a large adult population. In addition, using the quantile regression we studied the simultaneous effects of the intervention on several points (10^th^, 20^th^, 30^th^, 40^th^, 50^th^, 60^th^, 70^th^, 80^th^, and 90^th^ quantiles) of the BMI distribution. This is not possible with ordinary least square (OLS) regression. In some cases, important information is lost concerning the behavior of the relationship in the extreme tails of the BMI distribution. This study has limitations that should be noted. Although families were randomly selected for the intervention and control groups, the inability to randomly allocate centers in the initial stage may have introduced selection bias. In addition, potential mediating and moderating factors in weight control, including the knowledge and attitude of the target population, as well as environmental and socio-economic factors, have not been evaluated. These unexamined variables could influence participation and engagement in healthy lifestyle interventions, as well as impact the understanding of the intervention’s mechanisms and the generalizability of the conclusions to all segments of the population. Similarly, as this was a multi-setting longitudinal study, it was impossible to determine the effect of each part of the intervention separately, which complicates efforts to refine and enhance the intervention methodology. Furthermore, the current intervention had no fidelity assessment protocol for the participating training sessions, which could affect the overall efficacy of the intervention. Also, since the research community was limited to an urban area, generalizing the results to the rural and suburban areas would be impossible.

## Conclusion

 The present study is the first to examine the effects of a practical and multi-setting lifestyle intervention on the full BMI spectrum in a middle-income country with different socio- cultural features from Western countries. Our results indicated the current intervention effectively reduced BMI in men at the initial follow-up and in women at a later follow-up, eventually benefiting all overweight women. However, the intervention’s impact did not persist in the long term. This finding can be useful for planning obesity prevention programs in communities with similar socioeconomic condition and highlights the importance of developing sustained and adaptable strategies for managing BMI transitions to combat overweight and obesity, emphasizing the need for continuous support to maintain long-term benefits. Future research should explore the long-term sustainability of intervention effects and investigate the influence of mediating factors on weight control. Additionally, studies in rural areas are needed to assess the generalizability of these findings.

## Competing Interests

 The authors declare that they have no competing interests.

## Consent for Publication

 Not applicable.

## Data Availability Statement

 The datasets used during the current study are available from the corresponding author on reasonable request.

## Ethical Approval

 This TLGS is registered at Iran Registry for Clinical Trials (IRCT), a WHO primary registry (identifier: IRCT138705301058N1, http://irct.ir; date: 29/10/2009). This study was approved by the Ethical Committee of Research Institute for Endocrine Sciences and the National Research Council of the Islamic Republic of Iran (Ethic No: 121). Informed consent was obtained from all individual participants included in the study. All procedures were in accordance with the ethical standards of the institutional and/or national research committee and with the 1964 Helsinki declaration and its later amendments or comparable ethical standards.
